# Socioeconomic inequality in abdominal obesity among older people in Purworejo District, Central Java, Indonesia – a decomposition analysis approach

**DOI:** 10.1186/s12939-017-0708-6

**Published:** 2017-12-12

**Authors:** Cahya Utamie Pujilestari, Lennarth Nyström, Margareta Norberg, Lars Weinehall, Mohammad Hakimi, Nawi Ng

**Affiliations:** 1 Unit of Epidemiology and Global Health, Department of Public Health and Clinical Medicine, Umeå University, 90187 Umeå, Sweden; 2 Centre for Reproductive Health, Faculty of Medicine, Gadjah Mada University, Yogyakarta, Indonesia

**Keywords:** Socio-economic inequality, Abdominal obesity, Decomposition analysis, Low- and middle-income countries, Older people

## Abstract

**Background:**

Obesity has become a global health challenge as its prevalence has increased globally in recent decades. Studies in high-income countries have shown that obesity is more prevalent among the poor. In contrast, obesity is more prevalent among the rich in low- and middle-income countries, hence requiring different focal points to design public health policies in the latter contexts. We examined socioeconomic inequalities in abdominal obesity in Purworejo District, Central Java, Indonesia and identified factors contributing to the inequalities.

**Methods:**

We utilised data from the WHO-INDEPTH Study on global AGEing and adult health (WHO-INDEPTH SAGE) conducted in the Purworejo Health and Demographic Surveillance System (HDSS) in Purworejo District, Indonesia in 2010. The study included 14,235 individuals aged 50 years and older. Inequalities in abdominal obesity across wealth groups were assessed separately for men and women using concentration indexes. Decomposition analysis was conducted to assess the determinants of socioeconomic inequalities in abdominal obesity.

**Results:**

Abdominal obesity was five-fold more prevalent among women than in men (30% vs. 6.1%; *p* < 0.001). The concentration index (CI) analysis showed that socioeconomic inequalities in abdominal obesity were less prominent among women (CI = 0.26, SE = 0.02, *p* < 0.001) compared to men (CI = 0.49, SE = 0.04, *p* < 0.001). Decomposition analysis showed that physical labour was the major determinant of socioeconomic inequalities in abdominal obesity among men, explaining 47% of the inequalities, followed by poor socioeconomic status (31%), ≤ 6 years of education (15%) and current smoking (11%). The three major determinants of socioeconomic inequalities in abdominal obesity among women were poor socio-economic status (48%), physical labour (17%) and no formal education (16%).

**Conclusion:**

Abdominal obesity was more prevalent among older women in a rural Indonesian setting. Socioeconomic inequality in abdominal obesity exists and concentrates more among the rich population in both sexes. The inequality gap is less prominent among women, indicating a trend towards obesity being more common in poor women. Policies to address social determinants of health need to be developed to address the socioeconomic inequality gaps in obesity, with particular focus on addressing the existing burden of obesity among the better-off population group, while preventing the imminent burden of obesity among the worst-off group, particularly among women.

## Background

The ‘Fair society and healthy lives’ report by Sir Richard Marmot revealed that people living in the poorest areas died seven years earlier than those living in the richest areas [1]. Recent studies show that risk factors such as smoking, hypertension, and obesity [2], non-communicable diseases (NCDs) [3] and deaths [4] were more prevalent among individuals in low socioeconomic groups. Tackling health inequalities is a priority of many health care systems globally [5].

The World Health Organisation (WHO) reported that the global prevalence of obesity doubled between 1980 and 2014 [6]. As many previous studies have shown a higher prevalence of obesity in high-income countries (HICs), obesity has been considered a HICs problem [7–11]. In the HICs, the overconsumption of food combined with sedentary work increases the risk of developing obesity [11]. In low-income countries (LICs), however, obesity has not been a threat in the past few decades due to food scarcity and predominantly laborious work with higher energy expenditure [12, 13]. In many low- and middle-income countries (LMICs), industrialisation and acceleration of urbanisation have increased the income and improved the economic level. This economic transition paves the path for nutrition transition with changes in diet patterns from traditional diets to modern diets. These transitions lead to the consumption of more energy-dense food and more sedentary physical activity at work, during leisure time and during transport, which consequently contributed to a significant increase in the prevalence of overweight and obesity in LMICs in the last decades [13–16].

The association between SES and obesity has been studied extensively in HICs [4, 8, 10]. A cross-sectional study among adults aged 15 years and older in Spain showed that obesity was concentrated among the poor [10]. A recent prospective cohort study of people aged 50 years and older in England also showed that obesity was more prevalent among the poor [4]. Studies in LMICs, in contrast, reported that better wealth and higher education were associated with overweight and obesity [13, 14, 17], indicating that obesity is more predominant among the rich. In addition, in several LMICs such as South Africa, Samoa and Indonesia, cultural factors and positive attitude towards obesity which perceive overweight and obesity as a sign of wealth and prosperity, have influenced the dynamics of the obesity epidemic [18–20].

In Indonesia, the prevalence of obesity has increased constantly during the last decades [17, 19, 21]. Repeated cross-sectional and panel studies using the Indonesian Family Life Survey (IFLS) data showed that the average body mass index (BMI) among the Indonesian population increased between 1993 and 2007 [19, 22]. As in many other countries [6, 8, 15], obesity was more pronounced among Indonesian women [17, 19, 23]. The increase in BMI was observed in all age groups and in both urban and rural areas. In 1993, the prevalence of obesity (BMI ≥ 25 kg/m^2^) among the older population age 45+ years was 14% in women and 8.5% in men. In 2007 the prevalence had increased to 31% in women and 17% in men [24]. The increasing BMIs were slightly higher in rural areas (from 20.5 to 21.4 among men and from 21.1 to 22.9 among women) than in urban areas (from 21.8 to 22.5 among men and from 22.8 to 23.9 among women) [19].

Although widely used in measuring and diagnosing obesity, the validity of BMI among older populations has been long debated. The debate focuses on changes in body composition among older people with visceral body fat, which accumulates more in the abdominal area [25], leading to abdominal obesity. Abdominal obesity refers to ectopic body fat stored in the abdomen [26], which might not be measured properly using BMI. Therefore, a number of studies have recommended the use of waist circumference in measuring adiposity and diagnosing obesity among older populations [25, 27, 28].

Understanding the burden of obesity by gender and socio-economic groups as well as determinants of the inequality of obesity burden between groups could contribute to the development of contextualised-appropriate public health policies in addressing the inequality gaps and prioritising actions in tackling obesity in Indonesia. To the best of our knowledge, studies on socioeconomic inequalities on abdominal obesity among older people are lacking. This study aims to fill this gap by exploring the socioeconomic inequalities in abdominal obesity and identifying socioeconomic determinants of the inequalities among men and women aged 50 years and older in Purworejo Districts, Central Java, Indonesia.

## Methods

### Study settings

This study was conducted in Purworejo district, Central Java province, Indonesia. The district is located in the southern part of Java Island with a population of 712.686 inhabitants in an area of 1035 km^2^, where 84% of the areas are agricultural land [29]. The district consists of 90% rural area and 10% small urban settlement, with the geographical terrains ranging from coastal in the south to hilly and mountainous in the north. A Health and Demographic Surveillance System (HDSS) site was established in Purworejo district in 1994, which became a member of the INDEPTH Network of HDSS sites in Africa and Asia in 1998. The Purworejo HDSS covers 55,000 individuals in 14,500 households in the district [30]. The HDSS collects the demographic data (birth, death, marital status, migration, etc.) on annual basis and household socioeconomic data every 5-year, plus ad-hoc surveys nested within the HDSS site.

### Data source

**Fig. 1:**
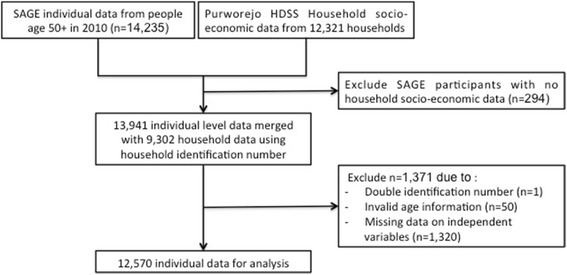
Flowchart of the study population generated from WHO-INDEPTH SAGE

We utilised data from the WHO-INDEPTH Study on global AGEing and adult health (WHO-INDEPTH SAGE) conducted in Purworejo HDSS in 2010. The survey included 14,235 individuals aged 50 years and older in the Purworejo HDSS (Fig. 1). Along with the WHO-INDEPTH SAGE survey, the socioeconomic census round was conducted among 12,321 households in the Purworejo HDSS. We linked the individual- and household-level data using the unique household identification number. In total, data from 13,941 individuals living in 9302 households were successfully merged. Due to missing data on key variables (mainly education and smoking status), a total of 1371 individuals were further excluded from the analysis; thus, the subsequent analyses were based on complete data from 12,570 individuals (88% of all respondents).

### Instruments and variables

The individual and household-level WHO-INDEPTH SAGE questionnaires [31] were translated into Indonesian, pilot-tested, and back translated into English to ensure the equivalence of the translation. The individual WHO-INDEPTH SAGE questionnaire contained self-reported information on respondents’ socio-demographic characteristics, health status, quality of life, ownership of health insurance and health care utilisation, health behaviours and risk factors, self-reported chronic conditions, self-reported weight and height, and waist circumference measurement [31].

The household questionnaire contained information on housing condition, infrastructure facilities, and ownership of assets. We selected some of the key from the household questionnaire to create the wealth index as a proxy for household SES using principal component analysis (PCA) [32]. All three assumptions of PCA (Kaiser-Meyer-Olkin measure of sampling adequacy, Bartlett’s test, and determinants of matrix correlation) [33] were tested and fulfilled. PCA generated the weight for each chosen asset and then created an index based on the sum of all weights of variables included in the PCA for each household. The index was categorised into SES quintiles with the 1st quintile representing the poorest group and the 5^th^ quintile representing the richest group.

We used data on household and individual socio-demographic characteristics (i.e. age, education, occupation, marital status, residence, and SES quintiles), smoking habits and self-reported chronic conditions as independent variables. Waist circumference was used as the dependent variable to measure abdominal obesity (Table 1).

**Table 1:** Operational definitions of the study variables

Variables	Categories and definitions
Dependent variable	
Abdominal obesity	- **Without abdominal obesity**: waist circumference < 90 cm for men or < 80 cm for women; - With abdominal obesity: waist circumference ≥ 90 cm for men or ≥ 80 cm for women [34, 57].
Independent variables	
Age group	**50–59**, 60–69, 70–79, 80+ years.
Education	- No formal education: never having any formal education; - ≤ 6 years: not completed elementary school, completed elementary school; - **> 6 years**: completed junior high school, high school, academy or university, master degree.
Occupation	- **Non-physical labour**: government worker, non-government worker, self-employed; - No occupation: retired, housewife, not-having a job; - Physical labour: farmer, fishermen, driver, rickshaw driver.
Marital status	- Single/widowed: not married, divorced, separated, widowed; - **Partnership**: married, living together.
Residence	**Coastal**, inland, hilly & mountainous
Wealth index	Composite index created using PCA and grouped into quintiles: 1^st^ (poorest), 2^nd^ (poor), 3^rd^ (middle), 4^th^ (rich), **5** ^**th**^ **(richest)**.
Smoking	- **Non-current smokers**: never smokers, ex-smokers - Current smokers: currently smoked daily or non-daily.
Self-reported chronic disease	- No: did not report having any chronic disease; - **Yes**: reported having at least one (≥ 1) of the chronic conditions including: hypertension, diabetes, stroke, cardiovascular disease (CVD), chronic obstructive pulmonary disease (COPD), asthma, and cancer.

Note: Words in bold in the 2^nd^ column refer to reference categories of each of the variable

### Data collection

A total of 25 field surveyors performed face-to face interviews under the supervision of four supervisors. All field workers participated in several training sessions in December 2009, during which the study protocols were discussed in detail and role-plays of interviews were conducted. Following the interview, waist circumference was measured at the point midway of the last palpable rib and top of iliac crest using a non-elastic measuring tape (in centimetres) [34]. The data were collected on paper forms, which were later scanned optically. Two data operators were responsible for validating the digital data and ensuring the safe storage of the databases.

### Data analyses

We used concentration index (CI) to show the concentration of abdominal obesity distribution in subgroups of populations across the wealth index. The CI ranges from −1 to +1. A negative CI indicates that abdominal obesity is concentrated among the disadvantaged (poor/deprived); a positive CI indicates that abdominal obesity is concentrated among the advantaged (rich/wealthy) and a zero CI suggests no inequality [35]. The concentration curve illustrates the CI visually. On the horizontal axis, the curve ranks the population from the most-disadvantaged to the most-advantaged group. The vertical axis indicates the cumulative percentage of abdominal obesity. The 45° diagonal line is called the line of equality [35], which represents perfect equality (i.e. CI = 0) in wealth distribution.

Decomposition analysis was performed to identify factors (covariates) contributing to the socioeconomic inequality in abdominal obesity [35]. The analysis decomposes the abdominal obesity CI into the explained component and the unexplained component. For the explained component, the analysis produces the elasticity, the concentration index (CI), and contribution to the CI for each covariate. The elasticity (frequency weighted coefficient) indicates the direction (positive or negative) and the degree of association (the impact) between the covariates and abdominal obesity [36]. Negative elasticity means the abdominal obesity would be lower in that category compared to the reference category (base). The contribution of CI was calculated by multiplying the elasticity and the CI. Thereafter, the percentage contribution of CI was calculated by dividing the contribution by the overall CI. Positive contribution in any covariate means the inequality would be less if that covariate were not present. The unexplained component remains as ‘residual’, which reflects the inequality that cannot be explained by the covariates included in the decomposition analysis [35, 36].

Analysis with binary health variables required an additional step of Wagstaff normalization in the CI and decomposition analysis as the minimum and maximum possible value of CI is not in the range of −1 and +1 [35, 37]. Normalization is needed to ensure that the CI is quantified in the range of −1 and +1, by dividing the CI by 1 minus the mean [35, 37]. To normalize the decomposition analysis, marginal effects from a probit regression were used in the elasticity calculation [35]. The affluent (advantaged) covariate was selected as the reference category (base). All analyses were conducted using Stata Version 13.

## Results

A total of 6788 women and 5782 men were included in the analysis. The median age was 62 (range 50–104) among men and 63 (range 50–109) among women. Table 2 shows the main socioeconomic characteristics of the participants, as well as mean waist circumference and abdominal obesity prevalence by socio-economic characteristics.

**Table 2:** Sociodemographic characteristics and mean waist circumference and prevalence of abdominal obesity in men (*n* = 5782) and women (*n* = 6788) by sociodemographic variables in the study

Variables	Percentage (%)			Mean waist circumference (SE)				Abdominal obesity prevalence (%)			
	Men	Women	*p* ^*^	Men	*p* ^**^	Women	*p* ^**^	Men	*p* ^***^	Women	*p* ^***^
Age (years)											
50–59	43.1	40.7	0.036	76.8 (0.18)	< 0.001	77.2 (0.2)	< 0.001	9.1	< 0.001	36.9	< 0.001
60–69	26.9	28.5		74.7 (0.21)		75.2 (0.23)		4.8		30.3	
70–79	22.1	23.0		73.0 (0.22)		73.1 (0.24)		3.7		21.5	
80+	7.9	7.8		72.7 (0.36)		72.4 (0.40)		2.2		19.1	
Education											
No formal education	10.6	31.1	< 0.001	72.5 (0.26)	< 0.001	72.9 (0.20)	< 0.001	1.3	< 0.001	20.6	< 0.001
≤ 6 years	64.9	56.8		73.9 (0.12)		75.3 (0.16)		3.4		30.3	
> 6 years	24.5	12.1		79.3 (0.26)		81.3 (0.39)		15.6		53.3	
Occupation											
Non-physical labour	10.1	9.7	< 0.001	80.8 (0.43)	< 0.001	79.0 (0.42)	< 0.001	19.8	< 0.001	44.1	< 0.001
No occupation	16.0	40.9		76.6 (0.34)		76.0 (0.21)		11.2		33.1	
Physical labour	73.9	49.4		73.9 (0.11)		74.0 (0.16)		3.3		24.8	
Marital status											
Single/widowed	12.7	40.4	< 0.001	73.2 (0.29)	< 0.001	74.2 (0.19)	< 0.001	3.4	0.001	25.6	< 0.001
Partnership	87.3	59.6		75.3 (0.12)		76.0 (0.16)		6.6		33.1	
Residences											
Coastal	49.8	49.8	0.119	75.2 (0.16)	< 0.001	75.7 (0.17)	< 0.001	6.5	< 0.001	31.4	< 0.001
Inland	23.9	25.2		76.2 (0.24)		76.3 (0.26)		9.2		35.4	
Hilly & mountainous	26.3	25.0		73.7 (0.19)		73.4 (0.23)		2.9		22.0	
Wealth index (quintiles)											
1st (poorest)	18.6	21.3	0.001	72.3 (0.21)	< 0.001	71.7 (0.23)	< 0.001	0.9	< 0.001	16.7	< 0.001
2nd	19.5	20.2		73.3 (0.21)		74.1 (0.26)		2.1		24.4	
3rd	20.0	19.6		74.4 (0.23)		75.7 (0.28)		4.9		32.4	
4th	21.1	19.4		75.8 (0.25)		76.2 (0.29)		7.1		34.6	
5th (richest)	20.8	19.5		79.2 (0.28)		79.2 (0.29)		15.0		43.8	
Smoking status											
Non-current smoker	25.0	96.8	< 0.001	77.1 (0.25)	< 0.001	75.4 (0.12)	< 0.001	11.2	< 0.001	30.6	< 0.001
Current smoker	75.0	3.2		74.3 (0.12)		70.1 (0.63)		4.5		15.3	
Self-reported chronic disease											
No	77.9	70.7	< 0.001	74.7 (0.12)	< 0.001	74.7 (0.14)	< 0.001	5.1	< 0.001	27.9	< 0.001
Yes	22.1	29.3		76.2 (0.26)		76.7 (0.24)		9.9		35.3	
Total				75.0 (0.11)		75.3 (0.12)	0.17	6.2		30.1	< 0.001

*SE =* standard error*; p* ^*^ *= p-*value for chi-2 test of difference between men and women; *p* ^**^ *= p-*value for ANOVA test of difference within sex group; *p* ^***^ *= p-*value for chi2 test of difference within sex group

Most men (75%) and women (88%) had less than 7 years of education (*p* < 0.001). About 74% of men and only 50% of women had a job involving physical labour, while 16% of men and 41% of women reported no occupation (most the women were housewives). Most men and women were in partnership, but the percentage of singles and widowed individuals was significantly higher in women (40% vs. 13%; *p* < 0.001). Self-reported chronic disease was significantly higher among women than men (29% vs. 22%; *p* < 0.001).

There was no difference in mean waist circumference between men and women (75.0 cm vs. 75.3 cm respectively, *p* = 0.17). However, abdominal obesity (defined as waist circumference ≥ 90 cm among men and ≥ 80 cm among women) was five-fold more prevalent among women (30.1% vs. 6.2%; *p* < 0.001). Among men, the prevalence of abdominal obesity was higher among those with > 6 years of education compared those with no formal education (16% vs. 1.3%; *p* < 0.001). The corresponding prevalence among women was 53% and 21%, respectively (*p* < 0.001). Abdominal obesity was significantly more prevalent among men and women who had a job involving no physical labour (20% among men and 44% among women, respectively) compared to those requiring physical labour (3.3% and 25%). The prevalence of abdominal obesity increased from the poorest to the richest group, from 17% to 44% in women and from 0.9% to 15% in men.

**Fig. 2:**
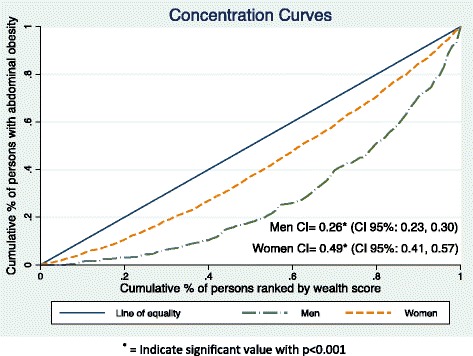
WHO-INDEPTH SAGE Purworejo study abdominal obesity concentration curve in men and women

The concentration index of abdominal obesity across wealth index was positive for both men (CI = 0.49, SE = 0.04, *p* < 0.001) and women (CI = 0.26, SE = 0.02, *p* < 0.001), as shown in the concentration curves with dashed lines below the line of equality (Fig. 2). The significant positive CI indicated that abdominal obesity was not equally distributed across the wealth index, but rather concentrated more among the rich population in both sexes. A lower CI value among women indicated less inequality observed among women compared to men.

Table 3 shows the results of the decomposition analysis, which include the elasticity, CIs and contribution of each covariate to the overall abdominal obesity inequality for men and women. The results showed that older men (80+ years) had a lower probability (negative elasticity) of having abdominal obesity compared to younger men (50–59 years). The abdominal obesity in older men (80+ years) was concentrated among the poor (CI: −0.26) and contributed positively (2.0%) to the abdominal obesity inequality. Men who lived in inland areas had a higher probability (positive elasticity) of having abdominal obesity compared to men who lived in coastal areas. In inland areas, abdominal obesity was concentrated among the rich men (CI: 0.06) and the contribution to the abdominal obesity inequality was small (0.4%).

**Table 3:** Decomposition of concentration indices for men and women in WHO-INDEPTH SAGE Purworejo study

Predictors	Men			Women		
	Elasticity	CIs	Contribution (%)	Elasticity	CIs	Contribution (%)
Age (years)						
50–59	Base	Base	Base	Base	Base	Base
60–69	−0.096	0.014	−0.001 (−0.2)	−0.033	−0.031	0.001 (0.4)
70–79	−0.086	−0.175	0.015 (3.1)	−0.076	−0.188	0.014 (5.5)
80+	−0.038	−0.261	0.009 (2.0)	−0.033	−0.101	0.003 (1.3)
Education						
No formal education	−0.045	−0.411	0.018 (3.8)	−0.139	−0.309	0.043 (16.4)
≤ 6 years	−0.268	−0.272	0.073 (14.8)	−0.197	0.006	−0.001 (−0.5)
> 6 years	Base	Base	Base	Base	Base	Base
Occupation						
Non-physical labor	Base	Base	Base	Base	Base	Base
No occupation	−0.005	0.203	−0.001 (−0.2)	−0.032	0.180	−0.005 (−2.2)
Physical labor	−0.577	−0.399	0.231 (46.8)	−0.155	−0.289	0.045 (17.1)
Marital status						
Single/widowed	−0.024	−0.142	0.003 (0.7)	−0.037	−0.106	0.003 (1.5)
Partnership	Base	Base	Base	Base	Base	Base
Residences						
Coastal	Base	Base	Base	Base	Base	Base
Inland	0.033	0.066	0.002 (0.4)	0.025	0.087	0.002 (0.8)
Hilly & mountainous	−0.033	−0.526	0.017 (3.6)	−0.021	−0.521	0.011 (4.2)
Wealth index (quintiles)						
1^st^ (poorest)	−0.108	−1.000	0.108 (22.0)	−0.099	−1.000	0.100 (37.9)
2^nd^	−0.086	−0.537	0.048 (9.4)	−0.059	−0.465	0.027 (10.5)
3^rd^	−0.039	−0.046	0.002 (0.4)	−0.019	0.033	−0.001 (−0.2)
4^th^	−0.027	0.473	−0.013 (−2.4)	−0.017	0.516	−0.009 (−3.5)
5^th^ (richest)	Base	Base	Base	Base	Base	Base
Smoking status						
Non-current smoker	Base	Base	Base	Base	Base	Base
Current smoker	−0.335	−0.154	0.052 (10.5)	−0.008	−0.322	0.002 (1.0)
Self-reported chronic disease						
No	−0.329	−0.069	0.023 (4.7)	−0.173	−0.048	0.008 (3.2)
Yes	Base	Base	Base	Base	Base	Base
		Residual	−0.093		Residual	0.016

The major contributors for abdominal obesity inequalities in men and women were occupational class, wealth status and education level. Among men, physical labour explained 47% of the socioeconomic inequality in abdominal obesity, followed by the poor and poorest wealth index quintile (31%), ≤ 6 years education (15%) and current smoking status (11%). Among women, the poor and poorest wealth index quintile explained 48% of the inequality, followed by physical labour (17%) and no-formal education (16%). Overall, the abdominal obesity inequality across wealth index in Purworejo district was explained by most of the covariates included in the study. This was confirmed by the small residuals both in men (−19%) and women (6.4%).

## Discussions

This study examines the socio-economic inequalities in abdominal obesity and the determinants of the inequalities among men and women aged 50 years and older in Purworejo Districts, Central Java, Indonesia. This study shows that socioeconomic inequality in abdominal obesity exists in both sexes, with abdominal obesity concentrated more among the rich. The major determinants of abdominal obesity inequality observed between poor and rich were wealth status, occupational class and level of education.

### Socioeconomic inequality in abdominal obesity

Our finding that abdominal obesity was concentrated more among the rich is consistent with the findings from other studies in Indonesia [17, 19, 20, 38, 39] and in other LMICs [13–15]. Obesity inequalities and its determinants reported in this study may be partly explained by economic and nutrition transition in Indonesia. The economic transition in Indonesia brings about the nutrition transition with changing diet patterns from traditional healthier staple food to energy-dense food [19, 38, 40]. Populations with higher socio-economic status changed their food consumption patterns earlier and faster than their poorer counterparts, as they have better accessibility and affordability to the foods. Studies have shown that they are less prone to food scarcity, and on the contrary, might have over-consumed food, often foods with high calorie contents [19, 38, 40, 41]. Therefore, they are at a higher risk of developing obesity compared to their poorer counterparts.

At the same time, economic development and technology advances also promote sedentary life across all wealth groups [13, 15, 19]. Developments in technology render work tasks less laborious and physically less demanding. Household appliances for performing household chores are now widely available and affordable [23]. The increased use of motorised private transportation also leads to less energy expenditure during transfer from one place to another [17, 20, 23, 42]. This low level of physical activity combined with a shift in food consumption patterns have been identified as major contributors to obesity in Indonesia [19].

### Social determinants of abdominal obesity

We observed a significant difference in abdominal obesity prevalence between older women and men. This finding is in line with other studies in Indonesia that showed a higher prevalence of obesity among adolescents and adults women [17], as well as in older adult women aged 45 years and older [24]. The gender difference could partly be explained by women’s physiology and metabolism during adolescence, pregnancy and menopause [6, 43]. Another potential explanation relates to studies of the long-term impact of early malnutrition that may affect energy intake and expenditure mechanism, appetite regulation and weight gain patterns differently in men and women. Studies showed that women who experienced childhood malnutrition were facing greater risk to be obese during their adulthood than men [16, 44–47]. In the 1980s, the estimated prevalence of stunting in Asia exceeded 60% [48]. We believe the condition in Indonesia was worse 50 years ago (1960s), especially among the young girls, as the newly independent country struggled from problems of malnutrition (mainly under-nutrition) and related infectious diseases.

This study also confirms a more prominent socio-economic inequality gap in abdominal obesity among Indonesian men compared to their female counterparts. Gender differences in the degree of inequality in this study appear to be inversely related to the differences observed in the prevalence of obesity. Women in all socio-economic groups are consistently more obese than men. This may indicate that the obesity epidemic has affected women in the poorer groups. An Asian Development Bank Institute (ADBI) study among Indonesian men and women aged 20+ showed that the concentration index of obesity (BMI ≥ 25) has decreased during the period of 1993 to 2014 (from 0.324 to 0.175 among men and from 0.178 to 0.038 among women), which might indicate the shift of the obesity burden to the poorer group. The study also showed that the concentration index was consistently lower among women, indicating that obesity is rapidly becoming the problem of poor women [49]. The same patterns have been observed in another study conducted in LMICs, with a shift of obesity burden among women from the rich to the poor, mainly in LMICs with a gross national income (GNI) per capita > 1000 US$ and with medium HDI [15].

Beyond the gender difference shown in obesity inequality in our study, we believed that education might have different roles in influencing the levels of obesity in men and women [50, 51]. In high-income countries, highly-educated women usually engage in a healthier lifestyle (e.g. regular physical exercise and healthier diet) [50, 51], more than their male counterparts with the same level of education. Studies in the US also showed that highly-educated women, but not men, were more likely to be dissatisfied with their body image and they prefer a thinner silhouette [51]. Our findings showed contrasting results in which that highly-educated older women have higher level of obesity than their male counterpart. Older women (50+) might no longer be concerned about their body image and therefore do not engage in routine physical exercise. A study of physical inactivity prevalence in five Asian countries among adults age 25–64 years old showed that Indonesian women were more physically inactive compared to men (26% vs. 12% with physical inactivity lifestyle), and respondents with higher education tended to be less active compared to their counterparts with a lower education level [52]. Nevertheless, when we looked into the level of obesity within men and women in the current study, it is shown that the obesity prevalence of men with > 6 years education was twelve times greater than for those with no formal education. Meanwhile among women the level of obesity between the highest and lowest education was only higher by two folds.

Other factors that might be related to inequality in obesity are social relationships (i.e. marriage) and cultural factors [53]. Several studies have suggested that marriage predicts weight gain in both men and women [17, 19, 23, 53]. In our study, even though marital status showed small contributions to socioeconomic inequality in abdominal obesity in both men and women, higher abdominal obesity prevalence was observed among those in partnerships. Averett et al. proposed four mechanisms to elucidate the relationship between marriage and obesity [54]. These four hypotheses included: selection (where the leaner individual are most likely to be selected into marriage), protection (where marriage will improve one’s health as social support increases and risky behaviour decreases), social obligation (where meals with richer and denser food will be served regularly as a marriage social obligation) and marriage market hypothesis (where those who are married may not maintain a leaner figure as they are no longer on the market) [54]. We hypothesise that older couples in our setting might have more regular meals with richer and denser foods, hence contributing to the larger waist measurements among married couples.

Cultural factors and population perception on obesity might influence the burden of obesity. In some LMICs, the population believes that larger body size reflects higher social strata [12, 19, 20]. Our qualitative study of the community’s perception of diabetes confirms this observation, in that the community believe that diabetes, which is also caused by obesity, is a disease of wealthy people [42]. Furthermore, another study using IFLS data also found that obese people in Indonesia were satisfied and happy with their lives compared to the non-obese [20].

## Strengths and limitations of the study

This study analyses abdominal obesity measured by waist circumference, which is superior to BMI, particularly among older people and Asian populations [34, 55]. Several studies have shown that waist circumference has a stronger association with type-2 diabetes, CVD, and all-cause mortality than those of BMI [55, 56].

Several limitations should be considered in interpreting our results. First, there were different recommendations on the cut-off values for waist circumference for Asian populations, which might hamper the comparability of our findings with those from other studies. We used the cut-offs of abdominal obesity for the Asian population recommended by the WHO and International Diabetes Federation (IDF) [34, 57]. Second, as we used binary outcome with obese and non-obese categories, the CI analysis needed to be corrected following the methods proposed by Wagstaff and the World Bank [35, 37]. This correction also facilitates the decomposition analysis. The use of other correction methods might result in slightly different estimates.

## Policy implications

This study indicates the importance of developing policies to address social determinants of health of obesity with particular focus on addressing the existing burden of obesity among the better-off population group, while preventing the imminent burden of obesity among the worst-off group, particularly among women.

### The need to reorient current policies on nutrition

The Indonesian government has continuously prioritised health promotion program to deal with severe malnutrition (underweight or stunting) in the national agenda [40]. Obesity, on the other hand, has received less attention from the government, as it has not been perceived as a public health threat [17, 19, 20]. The IFLS data during 1993–2007 showed a decrease in the prevalence of stunting in Indonesia while at the same time, the prevalence of overweight and obesity in children and adult populations increased significantly in both urban and rural settings [19, 38, 40, 58]. The socio-economic patterns of obesity in Indonesia start resembling the patterns observed in the HICs, where obesity is more common among the poor and less among the rich and well-educated, particularly among women [4, 8]. As one of the main risk factors for chronic diseases, obesity epidemic can imminently lead to a larger burden of chronic diseases and health-care expenditure [6]. Hence, it is very important for the Indonesian government to develop health promotion programme to address the double burden of malnutrition, with a specific focus to tackle the imminent obesity epidemic in Indonesia [38–40].

### Community-based health promotion strategies

The Indonesian government has implemented several national community movements such as health promotion programmes to promote physical activity and healthy eating in order to decrease the prevalence of obesity [59]. Public awareness of obesity and knowledge of its health risks need to be raised through health promotion activities. The healthcare providers at primary health care units need to actively educate communities, especially women, regarding the benefit of consuming healthier food (more consumption of fruits and vegetable, healthy fats, reduction of sugar and salt intake) and promoting physical exercise during leisure time (for example the existing exercise program called ‘*senam lansia*’ targeting older people in neighbourhood). Furthermore, changing the socially positive perception of obesity among the lay communities is also important, as it would be difficult to address obesity as a public health problem when being fat is still perceived as a reflection of prosperity [20]. These activities could be achieved through multiple channels, including mass media campaigns, which could hopefully change the lay perception of the cultural value of obesity. It is also essential to maintain and improve the community health post’s programme for NCDs called *POSBINDU PTM* (*‘pos pembinaan terpadu penyakit tidak menular’*), to detect the NCD risk factors including obesity, and address them at earlier stages (through routine measurement of height, weight, waist circumference, blood pressure, etc.) [60]. As these community health post programmes have good coverage in Indonesia, they have the potential to reach larger population groups, especially the poorer population.

### Strengthening the Indonesia’s poverty reduction programme

In 2010, the Indonesian government established a National Team for the Acceleration of Poverty Reduction called *TNP2K* (*Tim Nasional Percepatan Penanggulangan Kemiskinan*) aiming to close the socio-economic inequality gaps in the country. The poverty reduction programmes include among which equal and universal access to education and health care, cash transfers, subsidized rice to poor families, etc. These national programmes do not target the older population in particular. Recently, the High-Risk Elderly Social Assistance Program called *ASLURETI* (cash transfers scheme for older people i.e. social pension), which has been successfully implemented in Aceh Jaya district, Aceh Province, Indonesia [61, 62], was scaled-up at the national level. These national programmes, yet to be evaluated, could be an effective way to reduce health inequality among the older population in Indonesia. These programmes could serve as an entry point for national programme to reduce the burden of obesity, as nearly 50% of the cash transfers were spent on food (e.g. meat, rice) at household level [62].

Community education on the health effects of obesity and campaigns to increase community’s awareness on healthier food choice could piggyback in the national poverty reduction programmes. Government efforts to provide low-cost healthy meals and fresh and green products are also important to increase the availability of these healthier choices for low-income older peoples. Obesity prevention programmes at school-level might also be important and effective for instilling healthier lifestyle since early life in order to prevent obesity in adulthood.

## Conclusions

This study shows the higher prevalence of abdominal obesity among older women in a rural Indonesian setting. Abdominal obesity concentrates more among the rich population in both sexes, but the inequality gap between the rich and the poor is less among women, indicating a trend towards obesity being more common among the poor women. Policies to address social determinants of health need to be developed to address the inequality gaps in obesity with particular focus on addressing the existing burden of obesity among the better-off population group, while preventing the imminent burden of obesity among the worst-off group, particularly among women. A gender-sensitive policy should be developed to address the gender gaps in obesity observed in Indonesia.

## References

[1] Marmot M, Bell R (2012). Fair society, healthy lives. Public Health.

[2] Di Cesare M, Khang Y-H, Asaria P, Blakely T, Cowan MJ, Farzadfar F (2013). Inequalities in non-communicable diseases and effective responses. Lancet.

[3] Korda RJ, Paige E, Yiengprugsawan V, Latz I, Friel S (2014). Income-related inequalities in chronic conditions, physical functioning and psychological distress among older people in Australia: cross-sectional findings from the 45 and up study. BMC Public Health.

[4] Demakakos P, Biddulph JP, Bobak M, Marmot MG (2016). Wealth and mortality at older ages: a prospective cohort study. J Epidemiol Community Health.

[5] Commission on Social Determinants of Health (2008). Closing the gap in a generation: health equity through action on the social determinants of health.

[6] World Health Organization (2014). Global status report on noncommunicable diseases 2014.

[7] Borrell LN, Samuel L (2014). Body mass index categories and mortality risk in US adults: the effect of overweight and obesity on advancing death. Am J Public Health.

[8] Devaux M, Sassi F (2013). Social inequalities in obesity and overweight in 11 OECD countries. Eur J Pub Health.

[9] Hajizadeh M, Karen Campbell M, Sarma S (2014). Socioeconomic inequalities in adult obesity risk in Canada: trends and decomposition analyses. Eur J Health Econ.

[10] Merino Ventosa M, Urbanos-Garrido RM (2016). Disentangling effects of socioeconomic status on obesity: a cross-sectional study of the Spanish adult population. Econ Hum Biol.

[11] Ng M, Fleming T, Robinson M, Thomson B, Graetz N, Margono C (2014). Global, regional, and national prevalence of overweight and obesity in children and adults during 1980–2013: a systematic analysis for the Global Burden of Disease Study 2013. Lancet.

[12] Sartorius B, Veerman LJ, Manyema M, Chola L, Hofman K (2015). Determinants of obesity and associated population attributability, South Africa: empirical evidence from a national panel survey, 2008–2012. PLoS One.

[13] Popkin BM, Adair LS, Ng SW (2012). Global nutrition transition and the pandemic of obesity in developing countries. Nutr Rev.

[14] Jones-Smith JC, Gordon-Larsen P, Siddiqi A, Popkin BM (2012). Is the burden of overweight shifting to the poor across the globe? Time trends among women in 39 low- and middle-income countries (1991–2008). Int J Obes.

[15] Dinsa GD, Goryakin Y, Fumagalli E, Suhrcke M (2012). Obesity and socioeconomic status in developing countries: a systematic review. Obes Rev.

[16] Ford ND, Patel SA, Narayan KM (2017). Obesity in low- and middle-income countries: burden, drivers, and emerging challenges. Annu Rev Public Health.

[17] Rachmi CN, Li M, Alison Baur L (2017). Overweight and obesity in Indonesia: prevalence and risk factors-a literature review. Public Health.

[18] Kanter R, Caballero B (2012). Global gender disparities in obesity: a review. Adv Nutr.

[19] Roemling C, Qaim M (2012). Obesity trends and determinants in Indonesia. Appetite.

[20] Sohn K (2017). The fatter are happier in Indonesia. Qual Life Res.

[21] Schroders J, Wall S, Hakimi M, Dewi FST, Weinehall L, Nichter M (2017). How is Indonesia coping with its epidemic of chronic noncommunicable diseases? A systematic review with meta-analysis. PLoS One.

[22] Vaezghasemi M, Razak F, Ng N, Subramanian SV (2016). Inter-individual inequality in BMI: An analysis of Indonesian Family Life Surveys (1993–2007). SSM Popul Health.

[23] Sari K, Mansyur M (2012). Female, live in urban, and the existence of a caregiver increased risk over-nutrition in elderly: an Indonesian national study 2010. Health Sci Indones.

[24] Witoelar F, Strauss J, Sikoki B, Smith JP, Majmundar M, National Research Council (US) Panel on Policy Research and Data Needs to Meet the Challenge of Aging in Asia (2012). Socioeconomic success and health in later life: evidence from the Indonesia Family Life Survey. Aging in Asia: Findings From New and Emerging Data Initiatives.

[25] Vlassopoulos A, Combet E, Lean ME (2014). Changing distributions of body size and adiposity with age. Int J Obes.

[26] Lee MJ, Wu Y, Fried SK (2013). Adipose tissue heterogeneity: implication of depot differences in adipose tissue for obesity complications. Mol Asp Med.

[27] Corona LP, Alexandre TD, Duarte YA, Lebrao ML (2017). Abdominal obesity as a risk factor for disability in Brazilian older adults. Public Health Nutr.

[28] Sohn K (2014). Sufficiently good measures of obesity: the case of a developing country. J Biosoc Sci.

[29] Badan Pusat Statistik Kabupaten Purworejo (2017). Kabupaten Purworejo dalam angka 2017 (Purworejo regency in figures 2017).

[30] Kowal P, Kahn K, Ng N, Naidoo N, Abdullah S, Bawah A (2010). Ageing and adult health status in eight lower-income countries: the INDEPTH WHO-SAGE collaboration. Glob Health Action.

[31] Kowal P, Chatterji S, Naidoo N, Biritwum R, Fan W, Lopez Ridaura R (2012). Data Resource Profile: The World Health Organization Study on global AGEing and adult health (SAGE). Int J Epidemiol.

[32] Howe LD, Galobardes B, Matijasevich A, Gordon D, Johnston D, Onwujekwe O (2012). Measuring socio-economic position for epidemiological studies in low- and middle-income countries: a methods of measurement in epidemiology paper. Int J Epidemiol.

[33] Vyas S, Kumaranayake L (2006). Constructing socio-economic status indices: how to use principal components analysis. Health Policy Plan.

[34] World Health Organization (2011). Waist circumference and waist–hip ratio: report of a WHO expert consultation, Geneva, 8–11 December 2008.

[35] O’Donnell O, van Doorslaer E, Wagstaff A, Lindelow M (2008). Analyzing health equity using household survey data : a guide to techniques and their implementation.

[36] Wagstaff A, van Doorslaer E, Watanabe N (2003). On decomposing the causes of health sector inequalities with an application to malnutrition inequalities in Vietnam. J Econom.

[37] Wagstaff A (2011). The concentration index of a binary outcome revisited. Health Econ.

[38] Roemling C, Qaim M (2013). Dual burden households and intra-household nutritional inequality in Indonesia. Econ Hum Biol.

[39] Hanandita W, Tampubolon G (2015). The double burden of malnutrition in Indonesia: social determinants and geographical variations. SSM Popul Health.

[40] Shrimpton R, Rokx C (2013). The Double Burden of Malnutrition in Indonesia.

[41] Razzaque A, Nahar L, Van Minh H, Ng N, Juvekar S, Ashraf A (2009). Social factors and overweight: evidence from nine Asian INDEPTH Network sites. Glob Health Action.

[42] Pujilestari CU, Ng N, Hakimi M, Eriksson M (2014). “It is not possible for me to have diabetes”-community perceptions on diabetes and its risk factors in Rural Purworejo District, Central Java, Indonesia. Glob J Health Sci.

[43] Pradhan AD (2014). Sex differences in the metabolic syndrome: implications for cardiovascular health in women. Clin Chem.

[44] Hernandez DC, Pressler E (2014). Accumulation of childhood poverty on young adult overweight or obese status: race/ethnicity and gender disparities. J Epidemiol Community Health.

[45] Case A, Menendez A (2009). Sex differences in obesity rates in poor countries: evidence from South Africa. Econ Hum Biol.

[46] van Abeelen AF, Elias SG, Roseboom TJ, Bossuyt PM, van der Schouw YT, Grobbee DE (2012). Postnatal acute famine and risk of overweight: the dutch hungerwinter study. Int J Pediatr.

[47] Wang Y, Wang X, Kong Y, Zhang JH, Zeng Q (2010). The Great Chinese Famine leads to shorter and overweight females in Chongqing Chinese population after 50 years. Obesity.

[48] de Onis M, Frongillo EA, Blossner M (2000). Is malnutrition declining? An analysis of changes in levels of child malnutrition since 1980. Bull World Health Organ.

[49] Aizawa T, Helble MC (2016). Socioeconomic inequity in excessive weight in Indonesia. ADBI Working Paper.

[50] Goldkamp J, Anderson S, Lifits-Podorozhansky Y, Gavard JA (2015). Women’s perceptions regarding obesity and comorbidities and provider interaction. J Obstet Gynecol Neonatal Nurs.

[51] Grogan S (2016). Body image: understanding body dissatisfaction in men, women and children.

[52] Ng N, Hakimi M, Van Minh H, Juvekar S, Razzaque A, Ashraf A (2009). Prevalence of physical inactivity in nine rural INDEPTH Health and Demographic Surveillance Systems in five Asian countries. Glob Health Action.

[53] Ball K, Crawford D, Crawford D, Jeffery R, Ball K, Brug J (2010). The role of socio-cultural factors in the obesity epidemic. Obesity epidemiology: from aetiology to public health.

[54] Averett SL, Sikora A, Argys LM (2008). For better or worse: relationship status and body mass index. Econ Hum Biol.

[55] Haldar S, Chia SC, Henry CJ (2015). Body composition in Asians and Caucasians: comparative analyses and influences on cardiometabolic outcomes. Adv Food Nutr Res.

[56] Howel D (2012). Waist circumference and abdominal obesity among older adults: patterns, prevalence and trends. PLoS One.

[57] Alberti KGMM, Zimmet P, Shaw J (2006). Metabolic syndrome—a new world-wide definition. A consensus statement from the International Diabetes Federation. Diabet Med.

[58] Rachmi CN, Agho KE, Li M, Baur LA (2016). Stunting, Underweight and Overweight in Children Aged 2.0–4.9 Years in Indonesia: Prevalence Trends and Associated Risk Factors. PLoS One.

[59] Kementerian Kesehatan Republik Indonesia. Rencana strategis kementerian kesehatan tahun 2015–2019 [National health planning, guidelines for year 2015–2019]. Jakarta, Indonesia; 2015. http://www.depkes.go.id/resources/download/info-publik/Renstra-2015.pdf. Accessed 15 Jun 2017

[60] Kementerian Kesehatan Republik Indonesia (2012). Petunjuk teknis pos pembinaan terpadu penyakit tidak menular (POSBINDU PTM) [Technical guidance for integrated health post for non-communicable diseases].

[61] Priebe J, Howell F (2014). Old-age poverty in Indonesia: empirical evidence and policy options – a role for social pensions, TNP2K Working Paper 07-2014.

[62] Tim Nasional Percepatan Penanggulangan Kemiskinan (TNP2K). Cash transfers for the elderly to address poverty and stimulate economic growth: an evaluation of Aceh Jaya’s old-age cash transfer. Jakarta, Indonesia; 2017. http://www.tnp2k.go.id/images/uploads/downloads/ASLURETI%20Summary_english_FINAL.pdf. Accessed 28 Sept 2017

